# Retraction: Role of glycogen synthase kinase-3β and PPAR-γ on epithelial-to-mesenchymal transition in DSS-induced colorectal fibrosis

**DOI:** 10.1371/journal.pone.0339816

**Published:** 2026-01-05

**Authors:** The PLOS One Editors

After this article [1] was published, concerns were raised about Figs 1, 2, 4, 6, 8, 9, 12, 13, S2, S4-S7, S9 and Table 2. Specifically,

- The following panels appear to partially or fully overlap:
  - Fig 1 H&E DSS in [1] and Fig 2A H&E DSS in [2] when flipped and rotated 90°.
  - Fig 1 Masson’s Trichrome H_2_O in [1] and Fig 2C in [3] when rotated 180°.
  - Fig 1 H&E DSS+GED in [1] and Fig 2A H&E DSS+GED in [2] when flipped.
  - Fig 2A Coll. I-III DSS+GED+GW in [1] and Fig 4A Collagen I-III DSS+GED+GW in [2].
  - Fig 2A Coll. I-III H_2_O+GED, Fig 6A H2O and Fig 4A SMAD-3 H_2_O+GED when flipped and rotated 90°.
  - Fig 2A Coll. I-III DSS in [1] and Fig 4A Collagen I-III DSS in [2] when flipped.
  - Fig 8 H_2_O and Fig 9 H_2_O when flipped and rotated 90°.
  - Fig S2 panel 5 and Fig S4 panel 5.
  - Fig S2 panel 6 and Fig S4 panel 6.
  - Fig S2 panel 8 and Fig S4 panel 7.
  - Fig S5 panel 1, Fig S7 panel 2, Fig 12B β-Actin and Fig 13C β-Actin.
  - Fig S5 panel 2, Fig S7 panel 1, Fig S9 panel 2 and Fig 13C β-catenin.
  - Fig S7 panel 4 and Fig S9 panel 4 at different exposures.
  - Fig S7 panel 5 and Fig S9 panel 5 at different exposures.
- In Fig S7 panel 1, the protein loading appears to be uneven, particularly in lane 4 where the band intensity appears different to the other lanes. In Fig S7 panel 2, lane 4 appears to show a band of greater intensity and therefore different loading conditions to Fig S7 panel 1. Fig S7 panels 1 and 2 appear to underly Fig 13C and therefore the associated quantitative data in Fig 13D should be interpreted with caution.
- There are multiple western blots in the SI Files of [1] that do not appear to match the indicated figure in [1], and multiple western blot panels in [1] that do not appear to have corresponding underlying blots in the SI Files of [1] and/or do not appear to have an associated experimental control in the SI Files of [1].
- There are several values in Table 2 in [1] that appear to be the same (when rounded) as values in Table 2 in [2]. Specifically, Colon weight (H_2_O, H_2_O+GED and DSS + GED + GW), Dilation (DSS + GED and DSS + GED + GW), Thickness (DSS + GED + GW), Adhesions (DSS, DSS + GED and DSS + GED + GW) and Total macroscopic score (DSS + GED + GW).
- Table 2 reports mouse colon lengths of less than 1 cm.
- The legends of Figs 12 and 13 do not appear to match the figures shown.

The corresponding author stated that the following panels in [1] are incorrect: Fig 1 H&E DSS, Masson’s Trichrome H_2_O and H&E DSS + GED, Fig 2A Coll. I-III DSS + GED + GW, Fig 6A H_2_O, Fig S5 panel 1, and Fig S7 panels 1 and 3–5. They also provided updated legends for Figs 12 and 13 and an updated Table 2.

The H&E DSS and H&E DSS + GED panels in Fig 1, Coll. I-III DSS + GED + GW and Coll. I-III DSS panels in Fig 2A, and Table 2 report material from [2], published in 2015 by Oxford University Press, Copyright © 2015 Crohn’s & Colitis Foundation of America, Inc. The H&E DSS and H&E DSS + GED panels in Fig 1, Coll. I-III DSS + GED + GW and Coll. I-III DSS panels in Fig 2A, and Table 2 are not offered under a CC BY license and are therefore excluded from this article’s [1] license.

In light of the nature and extent of the above concerns, which question the reliability and integrity of the reported results and conclusions, the *PLOS One* Editors retract this article.

JDG and VF did not agree with the retraction. AV responded but expressed neither agreement nor disagreement with the editorial decision. RS, SS, CD, PD, SP, LC, EG, and GL either did not respond directly or could not be reached.

JDG and VF apologize for the issues with the published article and stand by the article’s findings.
